# The roles of IL-17C in T cell-dependent and -independent inflammatory diseases

**DOI:** 10.1038/s41598-018-34054-x

**Published:** 2018-10-24

**Authors:** Sachiko Yamaguchi, Aya Nambu, Takafumi Numata, Takamichi Yoshizaki, Seiko Narushima, Eri Shimura, Yoshihisa Hiraishi, Ken Arae, Hideaki Morita, Kenji Matsumoto, Ichiro Hisatome, Katsuko Sudo, Susumu Nakae

**Affiliations:** 1 Laboratory of Systems Biology, Center for Experimental Medicine and Systems Biology, The Institute of Medical Science, The University of Tokyo, Tokyo, 108-8639 Japan; 2 Department of Dermatology, Tokyo Medical University, Tokyo, 160-0023 Japan; 3 Department of Cardiovascular Surgery, Saitama Medical Center, Jichi Medical University, Saitama, 330-8503 Japan; 4 Department of Chemistry, Juntendo University School of Medicine, Chiba, 270-1695 Japan; 5 Department of Immunology, Faculty of Health Sciences, Kyorin University, Tokyo, 181-8612 Japan; 6 Department of Allergy and Clinical Immunology, National Research Institute for Child Health and Development, Tokyo, 157-8535 Japan; 7 Division of Regenerative Medicine and Therapeutics, Tottori University Graduate School of Medical Science, Yonago, 683-8503 Japan; 8 Animal Research Center, Tokyo Medical University, Tokyo, 160-8402 Japan; 9 Precursory Research for Embryonic Science and Technology, Japan Science and Technology Agency, Saitama, 332-0012 Japan

## Abstract

IL-17C, which is a member of the IL-17 family of cytokines, is preferentially produced by epithelial cells in the lung, skin and colon, suggesting that IL-17C may be involved in not only host defense but also inflammatory diseases in those tissues. In support of that, IL-17C was demonstrated to contribute to development of T cell-dependent imiquimod-induced psoriatic dermatitis and T cell-independent dextran sodium sulfate-induced acute colitis using mice deficient in IL-17C and/or IL-17RE, which is a component of the receptor for IL-17C. However, the roles of IL-17C in other inflammatory diseases remain poorly understood. Therefore, we investigated the contributions of IL-17C to development of certain disease models using *Il17c*^−/−^ mice, which we newly generated. Those mice showed normal development of T cell-dependent inflammatory diseases such as FITC- and DNFB-induced contact dermatitis/contact hypersensitivity (CHS) and concanavalin A-induced hepatitis, and T cell-independent inflammatory diseases such as bleomycin-induced pulmonary fibrosis, papain-induced airway eosinophilia and LPS-induced airway neutrophilia. On the other hand, those mice were highly resistant to LPS-induced endotoxin shock, indicating that IL-17C is crucial for protection against that immunological reaction. Therefore, IL-17C neutralization may represent a novel therapeutic approach for sepsis, in addition to psoriasis and acute colitis.

## Introduction

IL-17C is a member of the IL-17 family of cytokines that includes IL-17A, IL-17B, IL-17D, IL-17E/IL-25 and IL-17F. It was originally identified as an IL-17-related cytokine by using EST databases^1,2^. It is well known that IL-17A and IL-17F are preferentially produced by immune cells such as T cells and group 3 innate lymphoid cells, while IL-17C and IL-25 are predominantly produced by non-immune cells such as colonic and lung epithelial cells^3–6^, keratinocytes^7,8^ and smooth muscle cells^9^, in mice and/or humans.

IL-17C is known to be important for host defense against such pathogens as *Pseudomonas aeruginosa*^10^. On the other hand, inappropriate/excessive IL-17C expression may be involved in development or regulation of various disorders in mice and/or humans. *Il17c* mRNA was reported to be increased in the inflamed skin from patients with psoriasis^7,11^ and in anti-TNF-induced psoriasiform skin lesions of patients with Crohn’s disease^12^. The mRNA for this cytokine is also increased in synovial fluid mononuclear cells from patients with rheumatoid arthritis^13^, and overexpression of IL-17C in mice resulted in exacerbation of collagen-induced arthritis^14^. SNPs in *Il17re* genes, which are components of the receptor for IL-17C^3,4^, were associated with risk for susceptibility to ulcerative colitis in Germany^15^. On the other hand, mice deficient in *Il17c* and *Il17re* showed aggravated inflammation during dextran sodium sulfate-induced colitis^3,4,16^, suggesting that IL-17C plays a regulatory role in the setting. Moreover, IL-17C may be involved in development of COPD^6^, cystic fibrosis^6^ and atherosclerosis^9^. IL-17C is also thought to be involved in tumorigenesis: increased expression of *Il17c* mRNA and IL-17RE protein was observed in lung cancer and hepatocellular carcinoma, respectively^17,18^, and tumor growth was reduced in *Il17c*-deficient (*Il17*c^−/−^) mice after non-typeable *Haemophilus influenza* infection^17^ and in *Il17re*^−/−^ mice on the *Apc*^min/+^ background^19^. IL-17C can enhance IL-17A production by Th17 cells, contributing to development of Th17 cell-mediated autoimmune diseases such as experimental autoimmune encephalomyelitis^20^.

In the present study, we investigated the roles of IL-17C in T cell-dependent diseases such as contact dermatitis and hepatitis, T cell-independent diseases such as pulmonary fibrosis and inflammation, and endotoxin shock using *Il17c*^−/−^ mice, which we newly generated.

## Results

### Generation of *Il17c*^−/−^ mice

**Figure 1:**
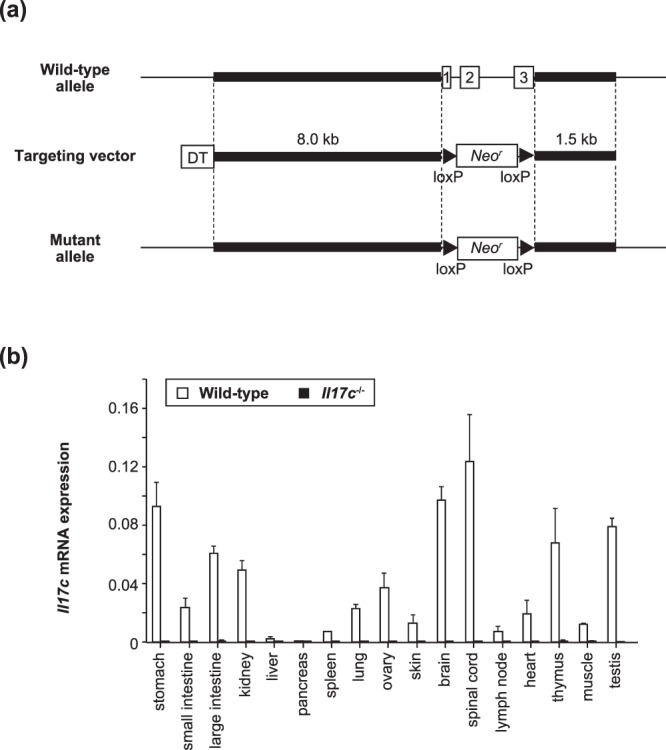
Generation of *Il17c*^−/−^ mice. (**a**) IL-17C gene targeting strategy. The region of the *Il17c* gene containing from exon 1 to exon 3 was replaced with a cassette containing a neomycin resistance gene (*Neo*^r^), flanked by *loxP* sequences. (**b**) Expression of *Il17c* mRNA in various tissues from wild-type (n = 3) and *Il17c*^−/−^ (n = 3) mice, determined by qPCR. The data show the mean + SEM.

*Il17c*^−/−^ mice were generated from C57BL/6N wild-type mice by replacing the *Il17c* genes with the neomycin resistance gene, flanked by *loxP* sequences in C57BL/6 mouse-derived ES cells (Fig. 1a). *Il17c*^−/−^ mice were born under specific-pathogen-free housing conditions at the expected Mendelian ratio, fertile, and did not show any gross phenotypic abnormalities (data not shown). Expression of *Il17c* mRNA was detected in various tissues from wild-type mice by quantitative PCR, whereas it was below the limit of detection in tissues from the *Il17c*^−/−^ mice (Fig. 1b). No apparent abnormalities were found in the proportions of immune cells in the thymus, LNs, spleen and/or bone marrow between the wild-type and *Il17c*^−/−^ mice (data not shown).

### IL-17C is not essential for development of T cell-dependent contact dermatitis

**Figure 2:**
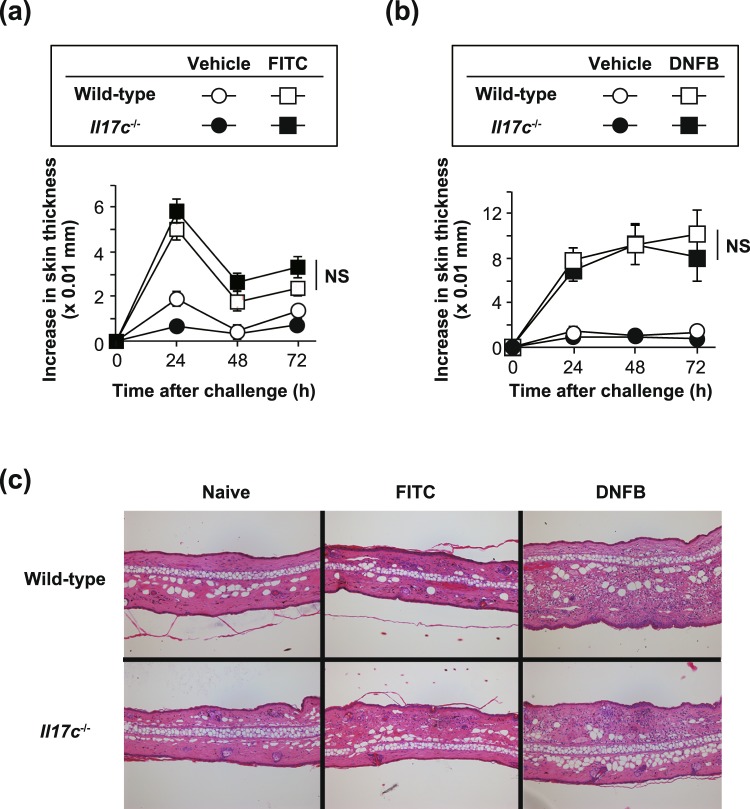
Normal development of T cell-dependent contact dermatitis in *Il17c*^−/−^ mice. (**a**) Wild-type (n = 18) and *Il17c*^−/−^ (n = 17) mice were epicutaneously sensitized and challenged with FITC or vehicle alone. The ear skin thickness of the mice was measured at the indicated time points after challenge. (**b**) Wild-type (n = 15) and *Il17c*^−/−^ (n = 17) mice were epicutaneously sensitized and challenged with DNFB or vehicle alone. The ear skin thickness of the mice was measured at the indicated time points after challenge. (**c**) Representative images of skin sections (from 24 hours in (**a**) and (**b**)) stained with hematoxylin and eosin. ×100. The data show the mean + SEM (**a** and **b**).

**Figure 3:**
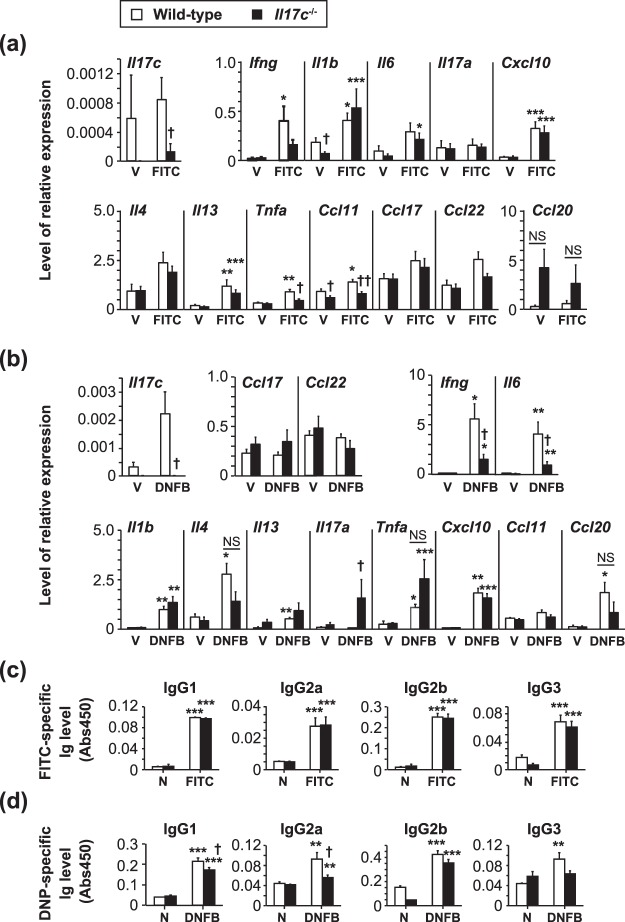
Gene expression in the skin, and Ab production in *Il17c*^−/−^ mice. (**a**) Relative mRNA expression levels of cytokines and chemokines in the ear skin of wild-type and *Il17c*^−/−^ mice 24 hours after FITC challenge. V = vehicle treatment. Wild-type mice (vehicle, n = 3; FITC, n = 5) and *Il17c*^−/−^ mice (vehicle, n = 3; FITC, n = 6). (**b**) Relative mRNA expression levels of cytokines and chemokines in the ear skin of wild-type and *Il17c*^−/−^ mice 24 hours after DNFB challenge. V = vehicle treatment. Wild-type mice (vehicle, n = 3; FITC, n = 5) and *Il17c*^−/−^ mice (vehicle, n = 3; FITC, n = 5). (**c**) Sera were collected from mice 72 hours after FITC challenge. The levels of FITC-specific Ig subsets in sera were determined by ELISA. N = naïve. Wild-type mice (naive, n = 8; FITC, n = 30) and *Il17c*^−/−^ mice (naive, n = 4; FITC, n = 32). (**d**) Sera were collected from mice 72 hours after DNFB challenge. The levels of DNP-specific Ig subsets in sera were determined by ELISA. N = naïve. Wild-type mice (naive, n = 8; DNFB, n = 27) and *Il17c*^−/−^ mice (naive, n = 5; DNFB, n = 30). The data show the mean + SEM. *p < 0.05, **p < 0.01 and ***p < 0.001: vehicle vs FITC or DNFB; and ^†^p < 0.05 and ^††^p < 0.01: wild-type mice vs. *Il17c*^−/−^ mice. Analyses by Mann-Whitney U-test, two-tailed (**a**, **b**) and Student’s t-test, two-tailed (**c**, **d**). NS = not significant.

It is known that hapten-induced contact dermatitis/contact hypersensitivity (CHS) is mediated by hapten-specific Th cells^21^. We found that the thickness of ear skin and severity of skin inflammation (based on histological analysis) were comparable between the *Il17c*^−/−^ mice and wild-type mice during FITC- and DNFB-induced CHS (Fig. 2a–c). However, mRNA expression for *Il17c* was increased in the ear skin from the wild-type mice, but not the *Il17c*^−/−^ mice, after FITC and DNFB challenge (Fig. 3a,b). In addition, mRNA expression for *Tnfa* and *Ccl11* in the skin after FITC challenge and mRNA expression for *Ifng* and *Il6* in the skin after DNFB challenge were reduced in the *Il17c*^−/−^ mice compared with the wild-type mice (Fig. 3a,b).

The levels of FITC-specific Ig subsets in sera were comparable between the *Il17c*^−/−^ and wild-type mice during FITC-induced CHS (Fig. 3c), whereas the levels of DNP-specific IgG1 and IgG2a, but not IgG3, in sera were significantly reduced in the *Il17c*^−/−^ mice compared with the wild-type mice during DNFB-induced CHS (Fig. 3d). These observations suggest that IL-17c is at least partially involved in such immune responses as mRNA expression and Ab production, but is not essential for development of CHS induced by FITC or DNFB.

### IL-17C is not required for development of T cell-mediated hepatitis

**Figure 4:**
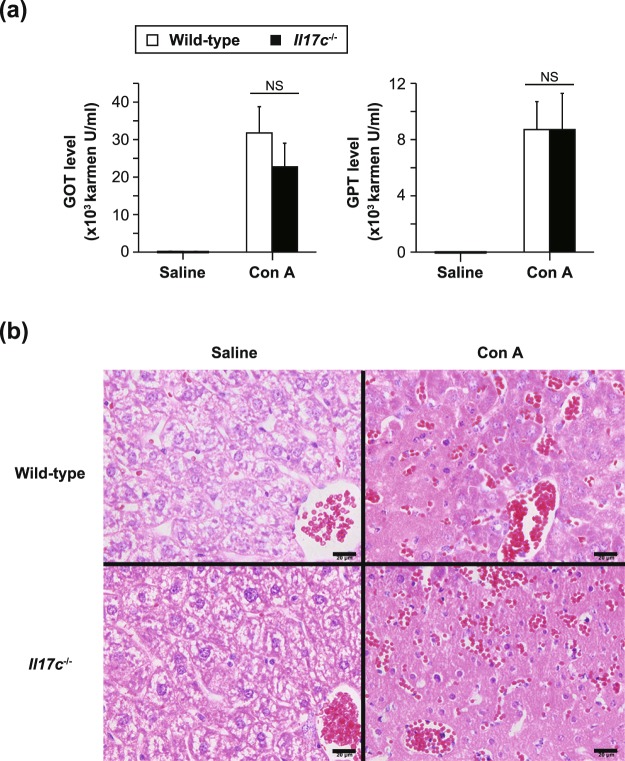
Normal development of T cell-dependent hepatitis in *Il17c*^−/−^ mice. Female mice were injected intravenously with concanavalin A (Con A). 24 hours later, sera and livers were collected. (**a**) Serum levels of GOT and GPT. Wild-type mice (Saline, n = 4; Con A, n = 5) and *Il17c*^−/−^ mice (Saline, n = 3; Con A, n = 5). The data show the mean + SEM. NS = not significant. (**b**) Representative images of liver sections stained with hematoxylin and eosin. Bars = 20 μm.

It is known that Con A-induced hepatitis is accompanied by apoptosis in tissues via Fas on T cells^22^. After Con A injection, the levels of GOT and GPT activities in sera were comparable in the *Il17c*^−/−^ and wild-type mice (Fig. 4a). In addition, infiltration of PMNs into the liver was also similarly observed in both groups (Fig. 4b). These observations suggest that IL-17C is not essential for T cell-mediated tissue injury in Con A-induced hepatitis.

### IL-17C is crucial for suppression of T cell-independent acute colitis

**Figure 5:**
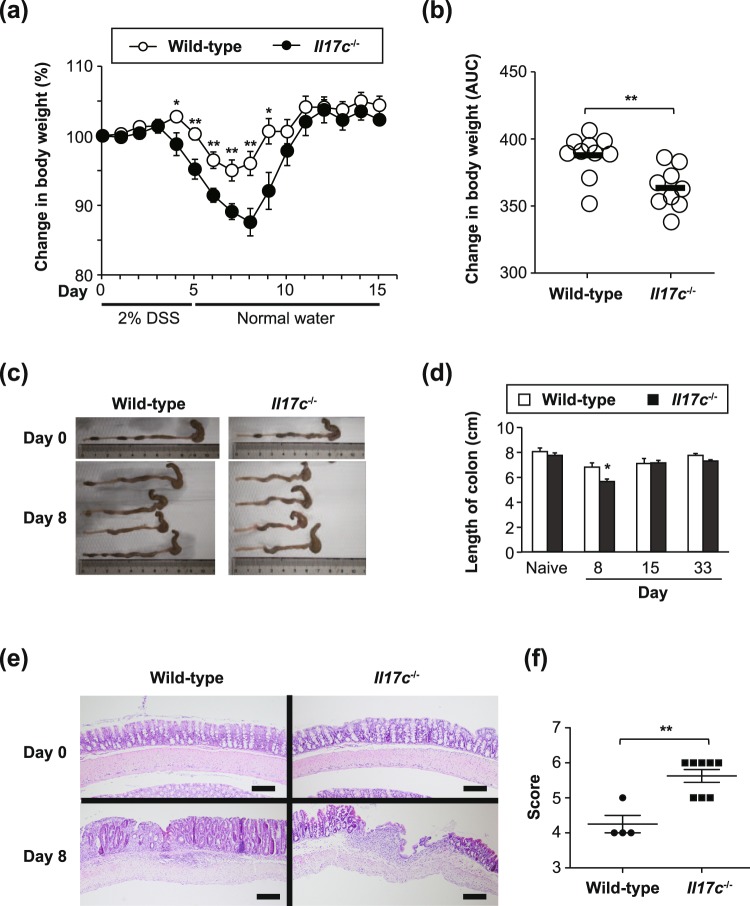
High susceptibility of *Il17c*^−/−^ mice to T cell-independent DSS-induced colitis. Female mice were provided sterile drinking water containing 2.0% DSS ad libitum for 5 days, followed by 15 days of regular drinking water. (**a**) Change in body weight (%). Wild-type mice (n = 10) and *Il17c*^−/−^ mice (n = 10). *p < 0.05 and **p < 0.01 vs. corresponding values for *Il17c*^−/−^ mice. (**b**) Area under the curve (AUC) for the body weight on Days 5–8 in (**a**). **p < 0.01. (**c**)Representative pictures of the animals’ colons on Day 8. (**d**) Length of colons on the indicated days. Wild-type mice (naïve, n = 4; and Days 8, 15, and 33, n = 4–5) and *Il17c*^−/−^ mice (naïve, n = 4; and Days 8, 15, and 33, n = 8–10). (**e**) Representative images of colon sections (Day 8) stained with hematoxylin and eosin. Bars = 100 μm. (**f**) Histological scores on Day 8. **p < 0.01. The data show the mean + SEM (**a**,**b**,**d**,**f**).

Dextran sodium sulfate (DSS)-induced acute colitis developed even in mice lacking T cells^23,24^. It was already demonstrated that *Il17c*^−/−^ mice and *Il17re*^−/−^ mice showed aggravated development of T cell-independent DSS-induced acute colitis^4,16^. We confirmed that body weight loss was more severe in the *Il17c*^−/−^ mice than in the wild-type mice after drinking of water containing DSS (Fig. 5a,b). The length of the colon in *Il17c*^−/−^ mice was significantly shortened compared with wild-type mice on Day 8 during DSS-induced colitis (Fig. 5c,d). Histological analysis revealed that local inflammation in the colon was more severe in the *Il17c*^−/−^ mice than in the wild-type mice on Day 8 during DSS-induced colitis (Fig. 5e,f). These observations indicate that IL-17C is crucial for suppression of DSS-induced acute colitis.

### IL-17C is not essential for development of T cell-independent lung diseases

**Figure 6:**
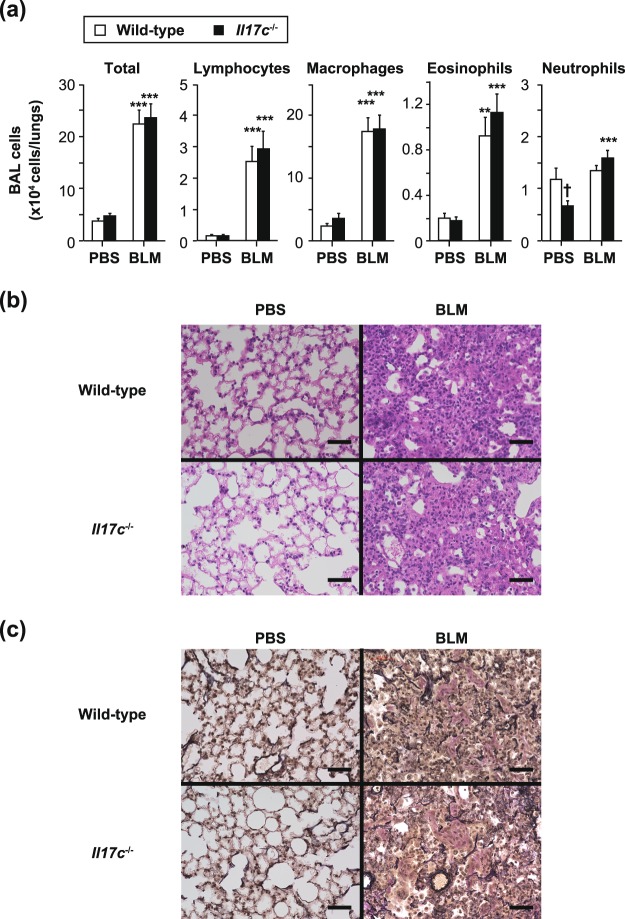
Normal development of T cell-independent pulmonary fibrosis in *Il17c*^−/−^ mice. Male mice were treated intratracheally with bleomycin (BLM) or PBS. 14 days later, bronchoalveolar lavage (BAL) fluids and the lungs were collected. (**a**) Cell counts in BAL fluids. Wild-type mice (PBS, n = 5; BLM, n = 9) and *Il17c*^−/−^ mice (PBS, n = 5; BLM, n = 7). The data show the mean + SEM. **p < 0.01 and ***p < 0.001: PBS vs BLM; and ^†^p < 0.05: wild-type mice vs. *Il17c*^−/−^ mice. (**b**,**c**) Representative images of lung sections. Hematoxylin and eosin staining (**b**) and Elastica van Gieson staining (**c**). Bars = 100 μm.

Constitutive expression of *Il17c* mRNA was observed in the lungs from wild-type mice, but not *Il17c*^−/−^ mice (Fig. 1b), suggesting that IL-17C may be involved in lung disease. Therefore, we investigated the role of IL-17C in bleomycin-induced pulmonary fibrosis, which develops independently of T cells^25^. However, the numbers of eosinophils, neutrophils, macrophages and lymphocytes in BAL fluids were comparable in *Il17c*^−/−^ and wild-type mice 14 days after intratracheal bleomycin challenge (Fig. 6a). Inflammation and fibrosis based on histological analysis (HE and EVG staining, respectively) were also comparable in the lungs of *Il17c*^−/−^ and wild-type mice during bleomycin-induced pulmonary fibrosis (Fig. 6b,c), indicating that IL-17C is not essential for development of bleomycin-induced pulmonary fibrosis.

**Figure 7:**
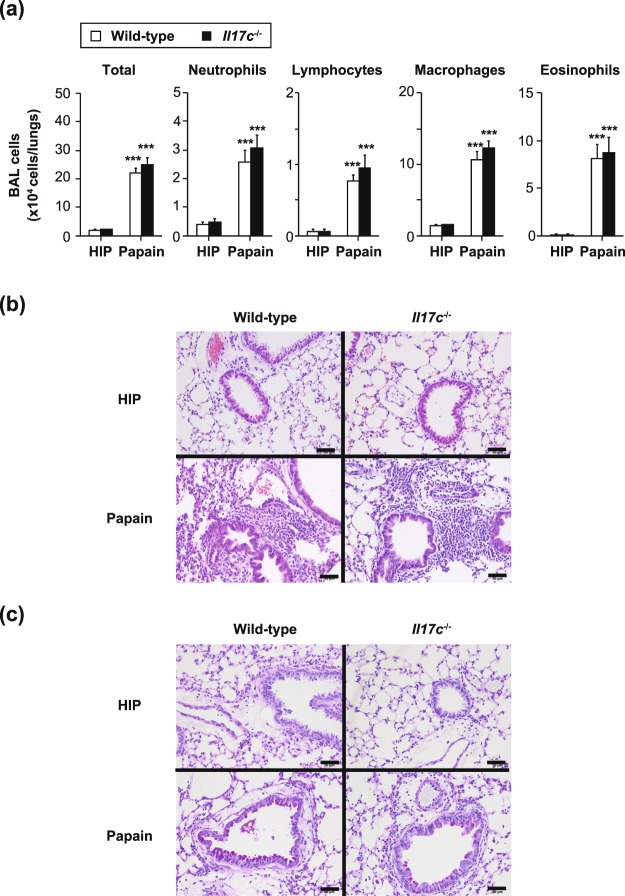
Normal development of T cell-independent airway eosinophilia in *Il17c*^−/−^ mice. Mice were treated intratracheally with papain or heat-inactivated papain (HIP). 24 hours after the last challenge, bronchoalveolar lavage (BAL) fluids and the lungs were collected. (**a**) Cell counts in BAL fluids. Wild-type mice (HIP, n = 10; Papain, n = 21) and *Il17c*^−/−^ mice (HIP, n = 10; Papain, n = 21). The data show the mean + SEM. **p < 0.01 and ***p < 0.001. (**b**,**c**) Representative images of lung sections stained with hematoxylin and eosin (**b**) and PAS (**c**). Bars = 50 μm.

Next, we investigated the role of IL-17C in development of airway eosinophilia induced by inhalation of papain, which is a papaya–derived cysteine and a homologue to house dust mite-derived Der p1 and human cathepsin B^26^. That eosinophilia induction is mediated by IL-33-stimulated ILC2s and basophils, but not T cells^27–30^. After papain inhalation, airway eosinophilia was assessed by the eosinophil count in BAL fluids and was similar in both *Il17c*^−/−^ and wild-type mice (Fig. 7a). The degree of pulmonary inflammation, based on histological analysis (HE and PAS staining, respectively), was also equivalent in the *Il17c*^−/−^ and wild-type mice during papain-induced airway eosinophilia (Fig. 7b,c). These observations suggest that IL-17C is not essential for induction of ILC2- and basophil-mediated papain-induced airway eosinophilia.

**Figure 8:**
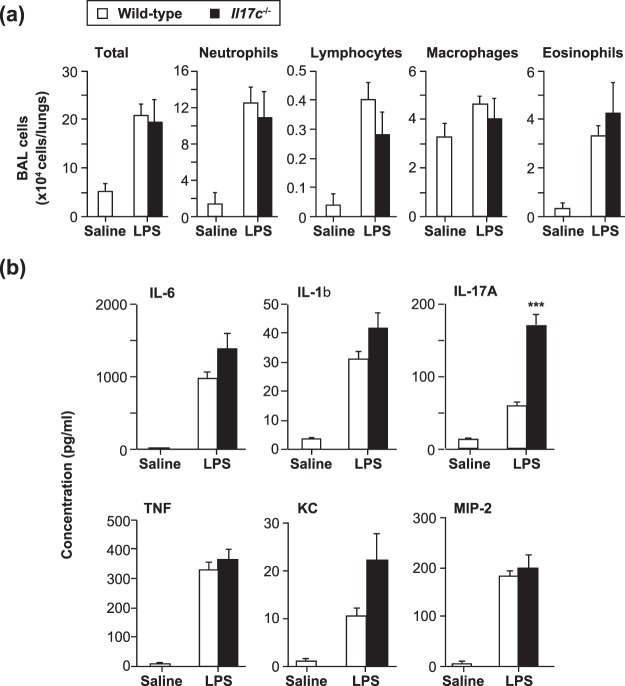
Normal development of T cell- and IL-17A-independent airway neutrophilia in *Il17c*^−/−^ mice. Mice were treated intratracheally with LPS or saline. 24 hours later, bronchoalveolar lavage (BAL) fluids were collected. (**a**)Cell counts in BAL fluids. Wild-type mice (saline, n = 5; LPS, n = 10) and *Il17c*^−/−^ mice (LPS, n = 5). (**b**) The levels of cytokines in the BAL fluids in (**a**). The data show the mean + SEM. ***p < 0.001 vs. wild-type mice.

Inhalation of LPS in mice resulted in induction of neutrophil-dominant airway inflammation^31^. In addition, LPS-induced airway neutrophilia developed normally in *Rag1*^−/−^ mice and *Il17a*^−/−^ mice^31^, indicating that it is T cell- and IL-17A-independent. We found that T cell- and IL-17A-independent LPS-induced airway neutrophilia was similar in *Il17c*^−/−^ mice compared with wild-type mice (Fig. 8a). The levels of IL-6, IL-1β and TNF in the BAL fluids were also comparable between *Il17c*^−/−^ and wild-type mice after LPS inhalation (Fig. 8b). Although the levels of IL-17A in the BAL fluids were significantly increased in *Il17c*^−/−^ mice compared with wild-type mice (Fig. 8b), these observations indicate that IL-17C is not essential for T cell- and IL-17A-independent LPS-induced airway neutrophilia.

### IL-17C is involved in induction of endotoxin shock

**Figure 9:**
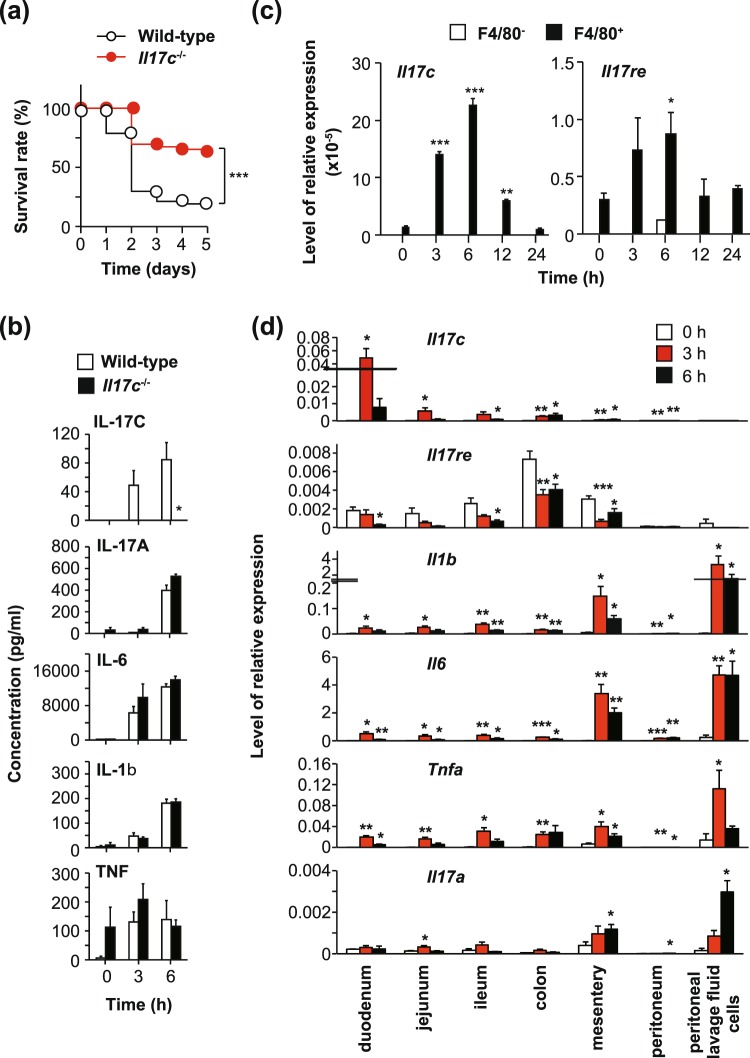
Strong resistance of *Il17c*^−/−^ mice to T cell-independent endotoxin shock. Female mice were injected intraperitoneally with LPS. After LPS injection, survival was monitored, and peritoneal fluids were collected at the indicated points. (**a**) Survival of wild-type (n = 47) and *Il17c*^−/−^ (n = 46) mice after LPS injection. ***p < 0.001. (**b**) The concentrations of IL-17C, IL-1β, IL-6, IL-17A and TNF in peritoneal lavage fluids from wild-type mice (0 h, n = 5; 3 h, n = 5; 6 h, n = 6) and *Il17c*^−/−^ mice (0 h, n = 5; 3 h, n = 5; 6 h, n = 6) were measured by ELISA. The data show the mean + SEM. *p < 0.05: wild-type mice vs. *Il17c*^−/−^ mice. (**c**) Peritoneal F4/80^−^ and F4/80^+^ cells were stimulated with LPS for the indicated times. The expression levels of *Il17c* and *Il17re* mRNAs in the cells were determined by quantitative PCR. The data show the mean + SEM (n = 3). (**d**) The expression levels of *Il17c*, *Il17re*, *Il1b*, *Il6*, *Il17a* and *Tnfa* mRNAs in the tissues and peritoneal lavage fluid cells from wild-type mice at 0, 3 and 6 h after LPS injection were determined by quantitative PCR. The data show the mean + SEM (n = 5). *p < 0.05, **p < 0.01 and ***p < 0.001 vs 0 h (**c**,**d**).

*Rag2*^−/−^ mice were more highly susceptible to LPS-induced endotoxin shock than wild-type mice, suggesting that T cells, B cells and/or NKT cells play suppressive roles in the setting^32^. In addition, looking at other members of the IL-17 cytokine family, IL-17A, but not IL-17F or IL-25, was important for LPS-induced endotoxin shock in mice^32^. However, the contribution of IL-17C to the response remains unclear. We found that the *Il17c*^−/−^ mice were more resistant to endotoxin shock after intraperitoneal LPS injection compared with the wild-type mice (Fig. 9a). The levels of IL-17C were increased in peritoneal lavage fluids from the wild-type mice, but not the *Il17c*^−/−^ mice, at 3 and 6 hours after intraperitoneal LPS injection (Fig. 9b). On the other hand, the levels of IL-17A, IL-6, IL-1β, TNF, KC and/or MIP2 were similarly increased in peritoneal lavage fluids of both groups and/or in sera (Fig. 9b and data not shown), suggesting that the effect of IL-17C on LPS-induced endotoxin shock is independent of those cytokines. In addition, the number of inflammatory cells and levels of IL-1β, IL-6, TNF, KC and MIP-2 in the BAL fluids were also identical between both groups in the setting (data not shown).

Next, we purified F4/80-negative and -positive cells from the peritoneal lavage fluid of naïve wild-type mice and cultured them in the presence and absence of LPS. After LPS stimulation, the expression levels of *Il17c* and *Il17re* mRNAs were significantly increased in F4/80^+^ cells but hardly detectable in F4/80-negative cells (Fig. 9c). However, the level of IL-17C protein in the culture supernatants of F4/80^+^ cells was below the limit of detection by ELISA (data not shown). In addition, the expression levels of *Il17c* mRNA were increased in the duodenum, jejunum, ileum and colon, but barely detectable in the peritoneum and peritoneal lavage fluid cells, of wild-type mice after LPS injection (Fig. 8d). On the other hand, *Il17re* mRNA was constitutively expressed in those tissues/cells, but its level decreased after LPS injection (Fig. 9d). In contrast to *Il17c* mRNA, expression of mRNA for each of *Il1b*, *Il6*, *Tnfa and Il17a* was increased in peritoneal lavage fluid cells from wild-type mice after LPS injection (Fig. 9d).

To elucidate the contribution of macrophage-derived IL-17C to macrophage activation, we cultured M-CSF-induced bone marrow cell-derived macrophages (BMMϕ) from wild-type and *Il17c*^−/−^ mice in the presence of LPS. The levels of IL-1β, IL-6 and TNF in the culture supernatants were similar between the two cell populations in the setting (data not shown). In addition, when peritoneal F4/80^+^ cells of wild-type mice were stimulated with LPS in the presence of recombinant IL-17C, LPS-mediated IL-1β, IL-6 and TNF productions were not affected (data not shown). These observations suggest that cells of the gut other than peritoneal F4/80^+^ cells are producers of and responders to IL-17C during LPS-induced endotoxin shock.

## Discussion

IL-17C is preferentially produced by non-immune cells such as colonic and lung epithelial cells^3–6^, keratinocytes^7,8^ and smooth muscle cells^9^, in mice and/or humans. It is known to enhance IL-17A production by Th17 cells, contributing to development of Th17 cell-mediated experimental autoimmune encephalomyelitis^20^. In the present study, we used *Il17c*^−/−^ mice to investigate the roles of IL-17C in T cell-dependent and -independent diseases.

We demonstrated that *Il17c* mRNA was constitutively expressed in the skin of mice (Fig. 1b), suggesting that IL-17C may be involved in host defense via the skin and/or in development of certain skin diseases. In support of that, *Il17c* mRNA was increased in inflamed skin from patients with psoriasis^7,11^. Moreover, *Il17c*^−/−^ and *Il17re*^−/−^ mice showed decreased development of imiquimod-induced psoriatic dermatitis^4^, which is mediated by IL-17-producing γδ T cells and ILC3, but not Th17 cells^33^.

Since IL-17C can enhance IL-17A production by Th17 cells^20^, we examined for involvement of IL-17C in development of allergic dermatitis, such as hapten-induced CHS, which is mediated by hapten-specific Th17 cells^21,34^. Indeed, we found that *Il17c* mRNA expression was increased in the inflamed skin of wild-type mice during FITC- and DNFB-induced CHS (Figs 2 and 3). Although mRNA expression for several cytokines and/or chemokines was reduced in the inflamed skin during FITC- and/or DNFB-induced CHS (Figs 2 and 3), *Il17c*^−/−^ mice showed normal development of FITC- or DNFB-induced CHS.

We previously reported that *Il17a*^−/−^ mice showed impaired DNFB-induced CHS^34^. Although IL-17A-heterozygous (*Il17a*^+/−^) mice show half–levels of IL-17A production compared with wild-type mice, the development of certain allergic diseases, including CHS, in *Il17a*^+/−^ mice was similar to in wild-type mice. These observations suggest that even the apparently reduced levels of IL-17A are sufficient to fully induce the inflammation of CHS in *Il17a*^+/−^ mice. We also found that *Il17c*^−/−^ mice showed reduced hapten-specific IgG1 and IgG2a production during DNFB-induced CHS. However, it was reported that B cell-deficient mice showed normal development of DNFB-induced CHS^35^. These observations suggest that although the deficiency of IL-17C resulted in reduced, but not completely abrogated, mRNA expression for certain cytokines and chemokines and Ab production, even the reduced mRNA levels may be sufficient for induction of inflammation. The levels of *Il1b* mRNA expression were comparable between the *Il17c*^−/−^ mice and wild-type mice during FITC- and DNFB-induced CHS (Fig. 3a,b). On the other hand, the levels of *Il1b* mRNA expression were reduced in the vehicle-treated skin of *Il17c*^−/−^ mice compared with the wild-type mice during FITC-, but not DNFB-, induced CHS (Fig. 3a,b). Those differences may have been caused by the different chemicals used to prepare the vehicles: FITC was dissolved in a mixture of dibutylphathalate and acetone, whereas DNFB was dissolved in a mixture of olive oil and acetone. Dibutylphathalate is known to be an irritant that can induce IL-1β-dependent activation of immune cells^36,37^. Therefore, although IL-17C seemed to be important for induction of *Il1b* mRNA expression in response to the irritant effect of dibutylphathalate, this cytokine is not essential for induction of *Il1b* mRNA expression in the elicitation phase of FITC-induced CHS. Thus, unlike the case of imiquimod-induced psoriatic dermatitis^4^, IL-17C is not essential for development of FITC- or DNFB-induced CHS. These observations suggest that IL-17C may be involved in IL-17-producing γδ T cell- and ILC3-mediated immune responses, rather than Th17 cell- and Tc17 cell-mediated immune responses, in the skin.

Moreover, it is known that liver injury during Con A-induced hepatitis is mediated by Fas on Tc cells^22^, and IL-17A is involved in the setting^38–40^. We demonstrated that Con A-induced hepatitis developed normally in *Il17c*^−/−^ mice, indicating that IL-17C is not essential for that development (Fig. 4). It was recently reported that Con A-induced hepatitis was milder in *Il17c*^−/−^ mice^41^. However, even when we used those authors’ experimental protocol, we found that Con A-induced hepatitis developed similarly in *Il17c*^−/−^ mice to in wild-type mice (data not shown). A similar discrepancy was also observed in *Il17c*^−/−^ mice during *Candida albicans* infection: Conti *et al*. reported that IL-17C was dispensable for host defense against *Candida albicans*^42^, whereas Huang *et al*. reported that IL-17C was important for that defense^43^.

The *Il17c*^−/−^ mice in the paper by Huang *et al*. were originally generated by using 129/TC1 ES cells, as they previously described^20^. In that earlier report^20^, the authors used C57BL/6 mice obtained from Jackson Laboratories. Although they did not describe how many times the *Il17c*^−/−^ mice on the 129/TC1 background were backcrossed with “C57BL/6J mice”, we presume that their mice must be on the “C57BL/6J background”. On the other hand, our *Il17c*^−/−^ mice were generated by using C57BL/6 ES cells and are on the “C57BL/6N” background. The susceptibility of mice to Con A-induced hepatitis was reported to be affected by their genetic background^44^. In addition, the intestinal microbiota, which affect immune responses, are different between C57BL/6J and C57BL/6N mice^45^. Moreover, susceptibility to Con A-induced hepatitis is influenced by the intestinal microbiota^46,47^. The apparent discrepancy between their and our results may be due to the different genetic backgrounds and/or housing environments of the mice.

*Il17c* mRNA was constitutively expressed in the lungs of mice (Fig. 1b), and lung epithelial cells are known to produce IL-17C, suggesting that IL-17C may be involved in host defense via the lungs and/or in development of certain pulmonary diseases such as COPD and cystic fibrosis^6^. *Il17a*^−/−^ mice showed reduced bleomycin-induced pulmonary fibrosis^48^, and it was suggested that Th17 cell- and γδ T cell-, but not iNKT cell-, derived IL-17A is important in those settings^48–50^. However, since bleomycin-induced pulmonary fibrosis is known to develop normally in T/B cell-deficient *scid/scid* mice^25^, IL-17A produced by non-immune/immune cells other than T cells may be crucial for that. Thus, it was not essential for development of bleomycin-induced pulmonary fibrosis (Fig. 6), although we expected IL-17C to contribute to development of T cell-independent IL-17A-mediated bleomycin-induced pulmonary fibrosis.

Like IL-17A, IL-17C is known to be involved in recruitment of neutrophils into local inflammatory sites^2,4,10,17^. Indeed, it was shown that IL-17A-mediated IL-17C expression by alveolar epithelial cells is important for recruitment of neutrophils by inducing IL-6 and neutrophil chemoattractants such as KC and MIP-2 in the lungs during *Pseudomonas aeruginosa* infection^10^. In contrast, we found that IL-17C is not essential for recruitment of neutrophils or production of cytokines (IL-1β, IL-6 and TNF) and chemokines (KC and MIP-2) in the lungs of mice after LPS inhalation (Fig. 8). Airway neutrophilia during *Pseudomonas aeruginosa* infection is dependent on IL-17A^10^, which is produced at least in part by Th17 cells^51^, whereas LPS-induced airway neutrophilia is independent of IL-17A and T cells^31^. Thus, IL-17C may be important for IL-17A-dependent, but not -independent, airway neutrophilia.

*Rag2*^−/−^ mice were highly susceptible, but *Il17a*^−/−^ mice were resistant, to LPS-induced endotoxin shock compared with wild-type mice^32^, suggesting that IL-17A produced by other non-immune/immune cells besides T and NKT cells is crucial for protection against LPS-induced endotoxin shock. Like *Il17a*^−/−^ mice^32^, we found that *Il17c*^−/−^ mice were resistant to T cell-independent IL-17A-mediated LPS-induced endotoxin shock, without any effect on production of IL-17A as well as other proinflammatory cytokines such as IL-1β, IL-6 and TNF (Fig. 9). Those findings suggest that IL-17C is crucial for protection against LPS-induced endotoxin shock, independently of IL-17A, IL-1β, IL-6 and TNF.

Taken all together, our observations indicate that IL-17C is not essential for development of T cell-dependent FITC- or DNFB-induced CHS or Con A-induced hepatitis. On the other hand, IL-17C is important for development of T cell-independent DSS-induced colitis and LPS-induced endotoxin shock, but not for bleomycin-induced pulmonary fibrosis, papain-induced airway eosinophilia or LPS-induced airway neutrophilia.

## Methods

### Mice

C57BL/6N wild-type mice were obtained from Japan SLC, Inc. *Il17c*^−/−^ mice on the C57BL/6N background were generated as follows. The *Il17* gene was disrupted by replacement of the region from exon 1 to exon 3 with a neomycin resistance gene, flanked by *loxP* sequences (Fig. 1a). Homologous regions were amplified by PCR using the following primers: 5′-AGTAACAACAGATACACCGCCACC-3′ and 5′-AACAGGTGAAGCCTGGCTGTGTGC-3′ to generate a 8-kb fragment, and 5′-TCCAGGACAGACATTCCAGGCACC-3′ and 5′-TAGGGACAGTAAGCAGCTTCAACC-3′ to generate a 1.5-kb fragment. The targeting vector was electroporated into C57BL/6N ES cells (EGR-101, kindly provided by Dr. Masaru Okabe, Osaka University). Male chimeric mice were obtained from two distinct targeted clones and mated with C57BL/6N female mice. Genotyping of *Il17c*^−/−^ mice was performed by PCR using the following primers: common (5′-CACAAGGCAAAGGCTGGGCATAGC-3′), WT (5′-CCAGCCCACCGAGCTCCGAGCTGC-3′) and MT (5′-GGAAGACAATAGCAGGCATGCTGG-3′). The common and WT primers were used for detection of wild-type alleles (~510 bp), and the common and MT primers were used for detection of mutant alleles (~270 bp). All mice were housed in a specific-pathogen-free environment at The Institute of Medical Science, The University of Tokyo. The animal protocols for experiments were approved by the Institutional Review Board of the Institute (A11-28 and A14-10), and all experiments were conducted according to the ethical and safety guidelines of the Institute.

### Quantitative PCR

Total RNA was prepared from tissues using Sepasol (Nacalai Tesque, Inc.) and treated with DNase (TURBO DNA-free-kit; Thermo Fisher Scientific Inc.). cDNA was synthesized from the isolated RNA by RT-PCR (PrimeScript RT Reagent kit; TAKARA BIO Inc.) or ReverTra Ace qPCR (RT Master Mix with gDNA; TOYOBO CO.). Quantitative real-time PCR was performed with SYBER Premix Ex Taq (TAKARA BIO Inc.) or THUNDERBIRD SYBR qPCR Mix (TOYOBO CO.) using a CFX384^TM^ Touch Real-time PCR Detection System (BioRad Laboratories, Inc.). Relative gene expression was determined against the expression levels of 18s rRNA (Fig. 1b) and *Gapdh* mRNA (other experiments). The designed primers are shown in Table 1.

**Table 1:** Sequences of primers.

Gene	Forward (5′–3′)	Reverse (5′–3′)
*Ccl11*	GAATCACCAACAACAGATGCAC	ATCCTGGACCCACTTCTTCTT
*Ccl17*	AGGAAGTTGGTGAGCTGGTATAAG	TCTTCACATGTTTGTCTTTGGG
*Ccl20*	GAGCTATTGTGGGTTTCACAAGAC	ACTCTTAGGCTGAGGAGGTTCAC
*Ccl22*	AGGTCCCTATGGTGCCAATGT	CGGCAGGATTTTGAGGTCCA
*Cxcl10*	CCAAGTGCTGCCGTCATTTTC	GGCTCGCAGGGATGATTTCAA
*Ifng*	GAACTGGCAAAAGGATGGTGA	TGTGGGTTGTTGACCTCAAAC
*Il1b*	CAACCAACAAGTGATATTCTCCATG	GATCCACACTCTCCAGCTGCA
*Il4*	TCCAAGGTGCTTCGCATATTTT	CAGCTTATCGATGAATCCAGGC
*Il6*	GAGGATACCACTCCCAACAGACC	AAGTGCATCATCGTTGTTCATACA
*Il13*	GGCAGCAGCTTGAGCACATT	GGCATAGGCAGCAAACCATG
*Il17a*	CCGCAATGAAGACCCTGATAGAT	AGAATTCATGTGGTGGTCCAGC
*Il17c*	CTGGAAGCTGACACTCACG	GGTAGCGGTTCTCATCTGTG
*Il17re*	CAGTCCCAGTGACGCTAGAC	ACCCACTAGAGCGGTGAGAG
*Tnfa*	GCCTCCCTCTCATCAGTTCT	CACTTGGTGGTTTGCTACGA
*Gapdh*	CCCACTCTTCCACCTTCGATG	AGGTCCACCACCCTGTTGCT
18s rRNA	GTAACCCGTTGAACCCCATT	CCATCCAATCGGTAGTAGCG

### Dextran sodium sulfate (DSS)-induced colitis

Mice were provided sterile drinking water containing 2% DSS (MW = 36,000-50,000; MP Biomedicals) ad libitum for 5 days, followed by 15 days of plain drinking water^27^. Each animal’s body weight was monitored daily. Histological scores were determined as described elsewhere^27^.

### Contact hypersensitivity (CHS)

FITC- and DNFB-induced CHS and measurement of FITC-specific and DNP-specific serum Igs were performed as described elsewhere^27^.

### Bleomycin-induced pulmonary fibrosis

Mice were treated intratracheally with 2 mg/kg bleomycin (NIPPON KAYAKU CO.) or an equal volume of PBS as a control. Fourteen days later, bronchoalveolar lavage (BAL) fluids were collected. The total cell counts and leukocyte profiles were determined with a hemocytometer (XT-1800i; Sysmex), as described previously^27^.

### Papain-induced airway inflammation

Papain-induced airway inflammation was generated as described elsewhere^27,30^. Twenty-four hours after the last inhalation of papain, BAL fluids were collected, and the total cell counts and leukocyte profiles were determined with a hemocytometer (XT-1800i; Sysmex), as above.

### LPS-induced airway inflammation

LPS-induced airway inflammation was established as described elsewhere^31^. In brief, mice were treated intratracheally with 20 μl of 0.5 mg/ml LPS (*Escherichia coli* serotype 0111:B4; Sigma–Aldrich) or an equal volume of saline as a control. Twenty-four hours after the last inhalation of LPS or saline, BAL fluids were collected, and the total cell counts and leukocyte profiles were determined as described above.

### Concanavalin A (Con A)-induced hepatitis

Con A-induced hepatitis was generated as described elsewhere^27^. Fourteen hours after the injection of Con A, sera were collected, and the levels of GOT and GPT in the sera were determined using Transaminase CII-test Wako (Wako).

### LPS-induced endotoxin shock

Mice were intraperitoneally injected with 15 mg/kg of LPS (*Escherichia coli* serotype 0111:B4; Sigma–Aldrich) as described elsewhere^27,32^. After LPS injection, the survival of the mice was monitored. Peritoneal cells and fluids were collected at 0, 3 and 6 hours after LPS injection, as described elsewhere^32^.

### ELISA for cytokines

The concentrations of IL-1β, IL-6, IL-17A, TNF, KC and MIP-2 were determined using ELISA kits (eBioscience, Biolegend or PeproTech), according to the manufacturers’ instructions. For IL-17C detection, plates (Nunc) for ELISA were coated with rat anti-mouse IL-17C mAb (MAB2306; R&D Systems; 2 μg/ml in PBS) as a capture Ab at room temperature overnight. After blocking with 1x Assay Diluent (BioLegend), samples and recombinant mouse IL-17C (R&D Systems) as a standard cytokine were applied to the wells, and the plates were incubated at 4 °C overnight. After washing the wells, goat anti-mouse IL-17C polyclonal Ab (AF2306; R&D Systems; 1.6 μg/ml in 1x Assay Diluent), as a detection Ab, was applied to the wells, followed by incubation at room temperature for 1 hour. After washing the wells, HRP-conjugated donkey anti-goat IgG (V805A; Promega; 1 μg/ml in 1x Assay Diluent) was added to the wells, followed by incubation at room temperature for 30 min. Tetramethylbenzidine solution (Nacalai Tesque) was used as the substrate. The reaction was stopped by adding 0.17 M sulfuric acid, and the OD450 was measured using a plate reader (VersaMax; Molecular Devices).

### Peritoneal cell culture

Peritoneal lavage fluid cells were collected from naïve wild-type mice. The cells were incubated with anti-CD16/CD32 mAb (93; eBioscience) in HBSS containing 2% FCS for 15 min at 4 °C, and then with biotinylated anti-mouse F4/80 mAb (BM8, BioLegend) for 25 min at 4 °C. After washing, the cells were incubated with streptavidin-beads (Miltenyi Biotec) for 15 min at 4 °C. After washing, F4/80-negative and -positive cells were separated using a MACS system (Miltenyi Biotec). The F4/80-negative and -positive cells were cultured in AIM V Medium (GIBCO) supplemented with AlbuMAX Supplement (GIBCO) in the presence of 2 μg/ml LPS for 0, 3, 6, 12 and 24 h at 37 °C. The cells were collected, and the total RNA of the cells was prepared as described above.

### Histology

Tissues were fixed in Carnoy’s solution and embedded in paraffin. Then sections were prepared and stained using hematoxylin and eosin, periodic acid-Schiff (PAS) or Elastica van Gieson (EVG) staining methods.

### Statistics

The Mann-Whitney U test, two-tailed (for quantitative PCR analysis), a Kaplan–Meier method (for survival) and a two-tailed, unpaired Student’s t-test (for other experiments) were applied to test for statistical significance using Graphpad Prism software (Graphpad Prism). P values of less than 0.05 were considered statistically significant.
